# PCANet based nonlocal means method for speckle noise removal in ultrasound images

**DOI:** 10.1371/journal.pone.0205390

**Published:** 2018-10-12

**Authors:** Houqiang Yu, Mingyue Ding, Xuming Zhang, Jinbo Wu

**Affiliations:** 1 Department of Biomedical Engineering, School of Life Science and Technology, Ministry of Education Key Laboratory of Molecular Biophysics, Huazhong University of Science and Technology, Wuhan, P.R. China; 2 School of Naval Architecture & Ocean Engineering, Huazhong University of Science & Technology, 1037 Luoyu Road, Wuhan, P. R. China; Texas A&M University, XX

## Abstract

Speckle reduction remains a critical issue for ultrasound image processing and analysis. The nonlocal means (NLM) filter has recently attached much attention due to its competitive despeckling performance. However, the existing NLM methods usually determine the similarity between two patches by directly utilizing the gray-level information of the noisy image, which renders it difficult to represent the structural similarity of ultrasound images effectively. To address this problem, the NLM method based on the simple deep learning baseline named PCANet is proposed by introducing the intrinsic features of image patches extracted by this network rather than the pixel intensities into the pixel similarity computation. In this approach, the improved two-stage PCANet is proposed by using Parametric Rectified Linear Unit (PReLU) activation function instead of the binary hashing and block histograms in the original PCANet. This model is firstly trained on the ultrasound database to learn the convolution kernels. Then, the trained PCANet is utilized to extract the intrinsic features from the image patches in the pre-denoised version of the noisy image to be despeckled. These obtained features are concatenated together to determine the structural similarity between image patches in the NLM method, based on which the weighted mean of all pixels in a search window is computed to produce the final despeckled image. Extensive experiments have been conducted on a variety of images to demonstrate the superiority of the proposed method over several well-known despeckling algorithm and the PCANet based NLM method using ReLU function and sigmoid function. Visual inspection indicates that the proposed method outperforms the compared methods in reducing speckle noise and preserving image details. The quantitative comparisons show that among all the evaluated methods, our method produces the best structural similarity index metrics (SSIM) values for the synthetic image, as well as the highest equivalent number of looks (ENL) value for the simulated image and the clinical ultrasound images.

## 1 Introduction

Medical imaging plays a critical role in disease monitoring and diagnosis. Compared with other imaging techniques such as X-ray, CT and MRI, ultrasound imaging is a noninvasive, real-time and radiation-free imaging modality. However, the ultrasound images are inevitably corrupted by speckle noise due to the coherent imaging mechanism from the scatters [1]. Such a noise reduces the sharpness of image details and complicates the diagnosis of the tiny structure of lesions. Therefore, despeckling is of great significance for improving ultrasound image quality. Moreover, as a pre-processing step, denoising will also benefit image post-processing tasks such as image segmentation, classification and registration.

In ultrasound imaging, speckle noise has a random granular pattern. The distribution of speckle noise is signal dependent and is governed by Fisher-Tippett distribution [2] or Gamma distribution [3], which can be represented [4, 5] as:

$$u(x,y)=v(x,y)+v{\left(x,y\right)}^{\gamma }\eta (x,y)$$ (Eq 1)

where (*x*,*y*) is the pixel location, *v*(*x*,*y*) is the clean image, *u*(*x*,*y*) is the noisy image, *η* is a Gaussian noise distribution with zero-mean and variance σ^2^, and the factor *γ* is related to the ultrasound devices and additional processing. Extensive studies indicate that *γ* = 0.5 can be used to model speckle noise in the ultrasound images [4, 6].

Many methods have been developed for ultrasound images despeckling [7]. In general, the existing despeckling methods can be classified as the spatial domain based methods and the frequency domain based ones. The traditional spatial domain based methods, such as Frost filter [8], Kuan filter [9], squeeze box filter (SBF) [10] and speckle reducing anisotropic diffusion (SRAD) filter [11], are based on the local comparison of pixels. One disadvantage of these approaches is that they cannot deliver sufficient noise reduction while preserving image details effectively. For the frequency domain based methods, the most popular techniques are the wavelet based methods [12–14]. These methods work by transforming speckle noise into additive noise and then removing it within the wavelet domain. They tend to introduce the artifacts related to the choice of mother wavelet.

Recently, the nonlocal means (NLM) filter proposed by Buades *et al*. [15] is considered as a state-of-the-art algorithm for eliminating the additive noise. This method explores the self-similarities between image patches instead of individual pixels, and each image pixel is restored by the weighted average of all pixels in a search window. Nowadays, the NLM method has been widely applied to denoise the medical images such as MR images and CT images [16–18]. Despite the success in removing Gaussian noise, the traditional nonlocal means (TNLM) method by its very nature becomes unsuitable for speckle noise reduction since speckle noise is different from Gaussian noise significantly. To overcome this drawback, several modified NLM-based approaches have been presented [5, 19–22] for despeckling. Specifically, Zhan *et al*. [5] have proposed a weight refining method for speckle noise reduction in which the weights are calculated in the lower-dimensional principal component analysis (PCA) subspace. Coupe *et al*. [19] have introduced the optimized Bayesian nonlocal means (OBNLM) filter. In contrast to the TNLM, the OBNLM filter determines the similarity between two image patches based on the Pearson distance derived by the Bayesian framework instead of the Euclidean distance of TNLM approach. Yang *et al*. [21] have presented a hybrid despeckling approach which combines the NLM with the local statistics of noise (NLMLS). In this method, the local statistics of speckle noise are used to pre-filter the ultrasound image and the similarity is computed based on the pre-filtered image to produce the final denoised result. Two total variation (TV) model based NLM methods have been proposed by Dong *et al*. [22] to reduce the multiplicative noise. Compared with the classical TV-based methods, the nonlocal TV methods perform better in preserving image textures and repetitive structures. The above-mentioned methods as the extension of TNLM method have contributed to the development of ultrasound image restoration techniques.

However, most of the NLM-based despeckling methods depend on the utilization of gray-level information and hand-crafted features. These features are not sufficient to accurately represent the structural similarity between image patches in the ultrasound images. If the intrinsic features can be learned from the images of interest for structural similarity computation, the better despeckled results can be obtained.

Deep learning, as a popular algorithm within the research community of machine learning, can automatically learn the intrinsic features from the training data. Up to now, various deep learning models, such as deep belief network (DBN), stacked auto-encode (SAE), convolutional neural networks (CNN) and nonlocal deep network [23], have been proposed. Among these models, the CNN is very popular due to the use of convolutional architectures. In recent years, many CNN-based variations have been introduced and successfully applied to various visual tasks such as feature extraction, classification [24, 25], super-resolution [26], object detection [27, 28] and image denoising [29–31]. In particular, Zhang *et al*. [31] have proposed a denoising convolutional neural network (DnCNN) model for removing the additive white Gaussian noise (AWGN), in which a very deep convolutional architecture is constructed while residual learning strategy and batch normalization are integrated to speed up the training process. Although the DnCNN and other CNN-based denoising models exhibit the promising denoised results, they generally involve the following problems. The training of CNN network is very complicated because it involves numerous parameters to be learned and requires too much empirical knowledge and special skills in parameter setting. In addition, most of the CNN-based denoisers are specially designed for denoising AWGN, and they cannot handle speckle noise very well. Thus, how to construct a simple and effective learning network for ultrasonic speckle reduction will pose a great challenge.

More recently, the principal components analysis network (PCANet), a very simple unsupervised deep learning baseline introduced by Chan *et al*. [32], has been proposed for image classification and hand-written digit recognition. This network consists of three simple data processing components: cascaded PCA, binary hashing and blockwise histograms. In this network, the basic PCA is first employed for learning the multistage convolution kernels, and the subsequent binary hashing and the blockwise histogram are utilized to produce the output features. Compared with the CNN-based models, the training of the PCANet model is extremely simple and efficient because no any regularized parameters or numerical optimization solvers are required for the involved filter learning [32].

Due to the advantage of the PCANet, it will be of significance to introduce this model into the NLM method to characterize structural similarity of image patches by extracting the robust intrinsic features from the ultrasound images. However, the binary hashing used in the PCANet may lead to the loss of useful feature information. To overcome the problem, we will present an improved PCANet in which an activation function Parametric Rectified Linear Unit (PReLU) [33, 34] is used instead of the binary hashing and block histograms at the output stage to extract the intrinsic features. To test the restoration performance of the proposed method, the extensive experiments on the synthetic image, the simulated image and the real ultrasound images are performed to make the comparisons among the proposed method and other despeckling methods. The experimental results demonstrate the superiority of the proposed method in speckle reduction and image detail preservation.

## 2 Methodology

### 2.1 The modified PCANet model

The PCANet model is a simple and valuable baseline for image classification and recognition tasks. The intrinsic features can be effectively extracted through three processing layers including the PCA-based convolutional layer, the binary hashing-based nonlinear layer and the histograms-based output layer. These processing methods used in the nonlinear layer and the output layer, which are similar to sparse representation strategies [35, 36], will result in the loss of image features. To overcome the disadvantage, we propose a modified PCANet, which consists of three processing components: the two convolutional layers and the output layer. The detailed structure of this network is given as follows.

#### A. The convolutional layer

Let the number of training images be *N*, the patch size be *k*_*1*_×*k*_*2*_ at all stages. For the *p*-th training image, all the patches around each pixel are collected step by step. For each patch in this training image, its mean is subtracted from the intensities of all pixels in this patch. Accordingly, the mean-removed matrix *A*_*p*_ = [*a*_*p*,*1*_,*a*_*p*,*2*_,…,*a*_*p*,*S*_] is produced for the whole image, where *a*_*p*,*s*_ denotes the *s*-th mean-removed vectorized patch, and *S* is the number of patches produced from the *p*-th image. By processing all the training images in the same way, the matrix *A* is obtained as:

$$A=[A_{1},A_{2},\cdots ,A_{N}]\in R^{k_{1}k_{2}\times SN}$$ (Eq 2)

The PCA operation is then implemented on *A* to derive the convolution kernels. The reconstruction error within a family of orthonormal filters is minimized by the PCA algorithm, i.e.,

$$\underset{}{\underset{V\in R^{k_{1}k_{2}\times L_{1}}}{\min}\|A-VV^{T}A{\|}_{F}^{2}},\;s.t.\;V^{T}V=I_{L_{1}}$$ (Eq 3)

where *L*_*1*_ is the number of filters in the first layer, $I_{L_{1}}$ is the identity matrix of size *L*_*1*_×*L*_*1*_, and ||·||_*F*_ denotes the Frobenius norm. The solutions of Eq (3) are the *L*_*1*_ principal eigenvectors of *AA*^*T*^. Therefore, the PCA filters are expressed as:

$$O_{l}^{1}=mat_{k_{1},k_{2}}(q_{l}(AA^{T}))\in R^{k_{1}\times k_{2}},\;l=1,2,\cdots ,L_{1}$$ (Eq 4)

where *q*_*l*_(*AA*^*T*^) represents the *l*-th principal eigenvector of *AA*^*T*^, and $mat_{k_{1},k_{2}}(q_{l}(AA^{T}))$ is the function that maps *q*_*l*_(*AA*^*T*^) to the matrix $O_{l}^{1}$.

Similar to the deep neural networks (DNNs), the multiple stages of PCA filters can be stacked for extracting higher level features. All outputs of the first convolutional layer are utilized as the inputs of the second convolutional layer. By repeating almost the same process as in the first convolutional layer, the leading *L*_*2*_ eigenvectors are obtained as the filters in the second one.

#### B. The output layer

For the *p*-th training image, there are *L*_*1*_ outputs ${\left\{F_{p,v}^{1}\right\}}_{v=1}^{L_{1}}$ from the first convolutional layer. Similarly, each input $F_{p,v}^{1}$ will produce *L*_*2*_ corresponding outputs ${\left\{F_{p,w}^{2}\right\}}_{w=1}^{L_{2}}$ from the second convolutional layer. In the original PCANet, all these *L*_*1*_×*L*_*2*_ outputs are binarized using the Heaviside step function while each group of *L*_*2*_ binary outputs is weighted summed to obtain *L*_*1*_ decimal-valued images. However, the previous analysis has shown that this operation will degrade the effectiveness of the extracted features. To ensure that all those features obtained from the input image can be kept as accurate as possible, the binary hashing and block histograms will be replaced by the PReLU function [34]. The reason of using PReLU function instead of ReLU and sigmoid functions is that it can help to preserve image details better by additionally utilizing the structural information carried by the negative entries. In this modified PCANet model, the PReLU will be utilized as the final output layer to map nonlinearity into the data to ensure the accuracy and robustness of the extracted intrinsic features. Here, the PReLU function is defined as:

$$\mathrm{PReLU}(x)-\{\begin{array}{l} x\;if\;x>0\\ ax\;if\;x\leq 0 \end{array}$$ (Eq 5)

When *a* = 0, the function degenerates into the Rectified Linear Unit (ReLU). If *a* is a very small fixed value, the PReLU will be reduced to the Leaky ReLU (LReLU). In this paper, *a* is fine-tuned as 0.25 to ensure the optimal despeckled results.

### 2.2 Feature extraction using the modified PCANet model

To realize robust intrinsic features extraction, the modified PCANet must be trained to obtain the convolution filter kernels. Considering that the PCANet uses the image patch based training strategy, we have chosen 200 general ultrasound images from the open ultrasound database [37] to train the PCANet. They consist of abdomen images, urinary tract images, pediatrics images, gynaecology images and musculo skeletal joints images, among which the number of each type of ultrasound images is 40. All the 200 images are cropped to be the size of 480×320 and pre-processed by the OBNLM filter.

**Fig 1:**
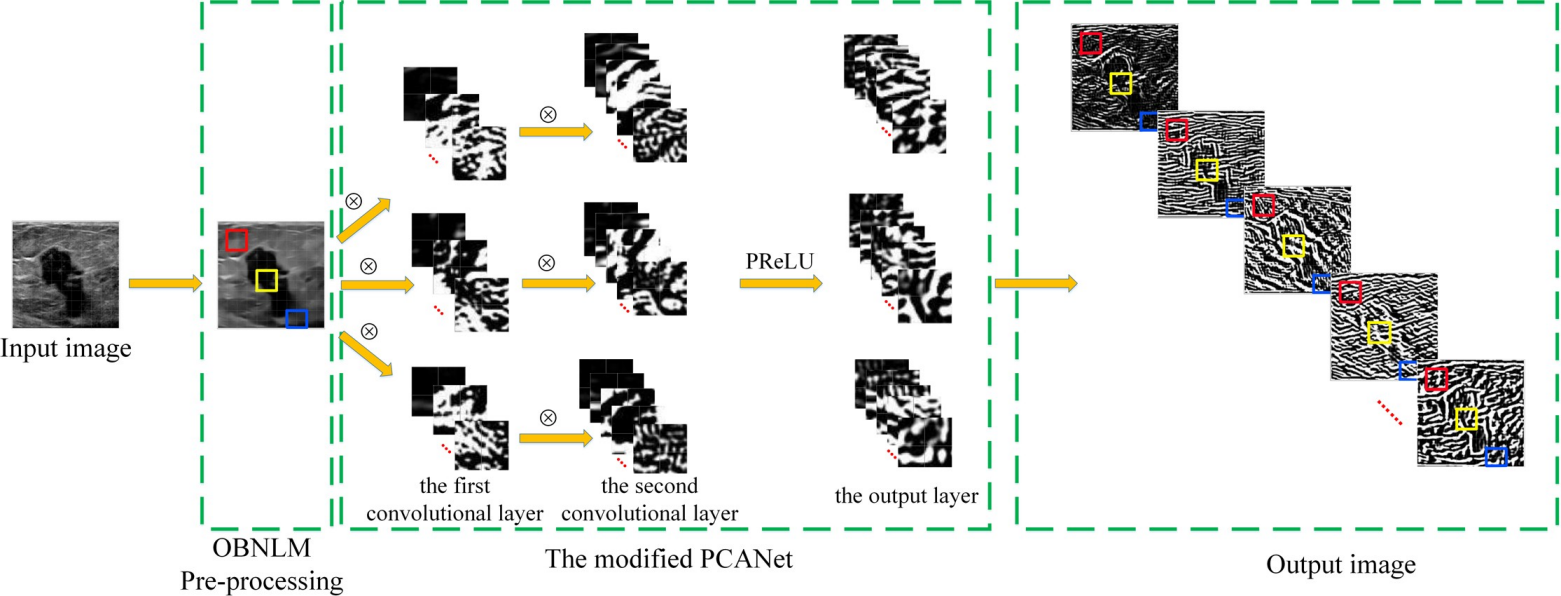
Illustration of feature extraction of image patches using the modified PCANet.

By using the trained PCA filters, the modified PCANet can extract the intrinsic features. A flowchart is given to illustrate how the modified PCANet extracts the features from the considered patches of an input image. As shown in Fig 1, the original noisy ultrasound image is pre-processed by the OBNLM filter before input into the PCANet. The patches in the pre-processed ultrasound image are convoluted with the trained PCA filters to produce *L*_*1*_ feature maps in the first convolutional layer. Then, each feature map is convoluted with the trained PCA filters to generate *L*_*2*_ feature maps in the second convolutional layer. All the *L*_*1*_×*L*_*2*_ feature maps are processed by the PReLU function to produce the final outputs.

### 2.3 The modified PCANet based nonlocal means method

**Fig 2:**
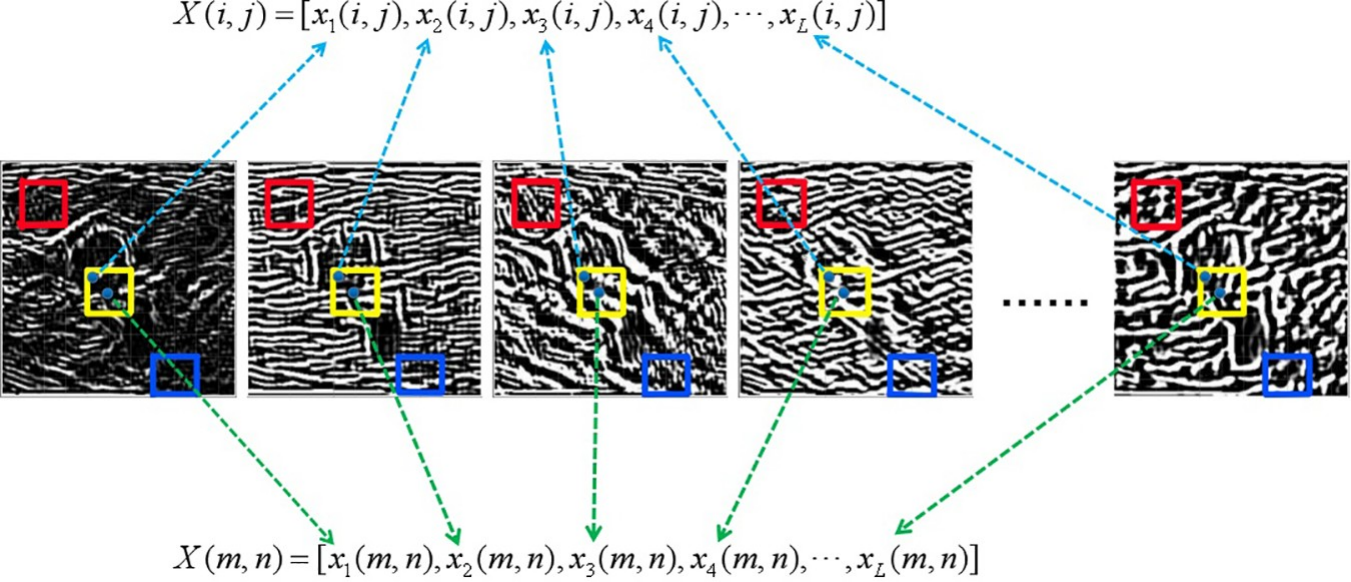
A detailed illustration of the construction of feature vectors based on the feature patches.

The main aim in this study is to improve the despeckling performance of NLM method. To refine the calculation of structural similarity between image patches in the NLM method, the proposed method exploits the robust intrinsic features extracted by the modified PCANet instead of the pixel intensities of the noisy image due to the strong feature learning abilities of PCANet. Fig 2 illustrates how to construct the feature vectors of size *L* for the computation of similarity weight, where *L* = *L*_*1*_×*L*_*2*_ is the number of feature images. The pixel intensities of the same location in a search window in all feature images are concatenated into a feature vector as shown in Fig 2. The structural similarity *ω*(*i*,*j*,*m*,*n*) between two image patches centered at (*i*,*j*) and (*m*,*n*) is computed based on the difference between the corresponding feature vectors, i.e.,

$$\omega (i,j,m,n)=e^{-\frac{\|X(i,j)-X(m,n){\|}_{2,\alpha }^{2}}{h^{2}}}$$ (Eq 6)

where *h* acts as a decay parameter controlling the degree of filtering, *X*(*i*,*j*) and *X*(*m*,*n*) are the concatenated feature vectors of image patches centered at (*i*,*j*) and (*m*,*n*), respectively. ||·||_*2*,*α*_ is a Gaussian weighted Euclidean distance with *α* denoting the standard deviation of Gaussian function.

Based on the structural similarity *ω*(*i*,*j*,*m*,*n*), the restored intensity *NLM*[*I(i*,*j)*] at (*i*,*j*) in the noisy image *I* is determined as:

$$NLM[I(i,j)]=\frac{\sum_{\left(m,n)\in \Omega (i,j\right)}\omega (i,j,m,n)I(m,n)}{\sum_{\left(m,n)\in \Omega (i,j\right)}\omega (i,j,m,n)}$$ (Eq 7)

where Ω(*i*,*j*) is the search window centered at (*i*,*j*).

To further improve the denoised results, the structural similarity between image patches will be refined by using the image restored by the proposed method instead of the pre-filtered results produced by the OBNLM method. The refined structural similarity will help the NLM filter to provide better despeckling performance.

### 2.4 Implementation of the proposed method

**Fig 3:**
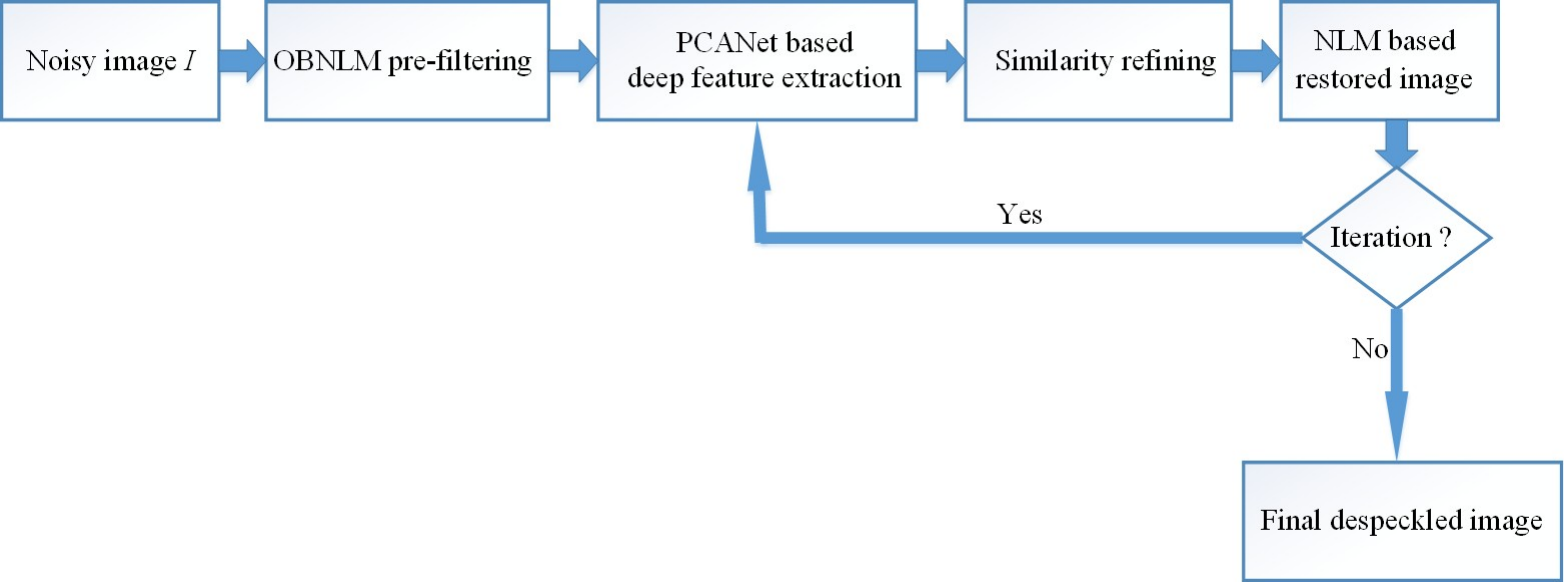
The scheme of the proposed method.

The implementation of the proposed method is summarized in Fig 3, the detailed description is as follows.

#### Step 1: Pre-processing

The original noisy image is pre-filtered by the OBNLM filter to produce the pre-processed image. The noise standard deviation is estimated, and the decay parameter is set as suggested in [38].

#### Step 2: Generation of the feature images

The pre-filtered image is input into the modified PCANet which has been trained using the open ultrasound database [37] to generate *L*_*1*_×*L*_*2*_ feature images as the outputs of this network.

#### Step 3: Computation of similarity weight

For each considered image patch in the original noisy image, the corresponding feature vector is constructed by using the feature images obtained in Step 2. The produced feature vectors are employed to compute the structural similarity between two image patches via Eq (6).

#### Step 4: Image restoration

Based on the similarity weight and the pre-fixed decay parameter, each pixel in the noisy image is restored by the NLM method based on Eq (7).

#### Step 5: Refinement of similarity weight

The restored image obtained in Step 4 is input into the modified PCANet to produce *L*_*1*_×*L*_*2*_ feature images, and then they are used to compute the structural similarity via Eq (6).

#### Step 6: Output of the final despeckled image

Based on the derived similarity weight in Step 5, the final despeckled image is produced by Eq (7).

## 3 Experimental results and discussion

In this section, the proposed method is tested on the synthetic image, the simulated image and the real ultrasound images. To demonstrate the superiority of the proposed PCANet based NLM method (PPCA-NLM), it will be compared with such traditional well-known despeckling algorithms as Frost, Kuan, SBF, SRAD, TNLM, OBNLM and NLMLS. Meanwhile, the proposed method will be also compared with the DnCNN method and the NLM methods using the original PCANet model (OPCA-NLM), the ReLU-based PCANet model (RPCA-NLM) and the sigmoid-based PCANet model (SPCA-NLM). In all experiments, the window size of Frost and Kuan filters is fixed to be 3×3. For SBF and SRAD filters, the parameters are fine-tuned as referred in [10, 11]. The sizes of similarity window and search window are set as 7×7 and 17×17 in the TNLM, OBNLM, NLMLS and PCANet based NLM filters, respectively. For the DnCNN denoiser, the same database is used as the PCANet to train the DnCNN model based on Eq (1), and the parameters and network structures are set as suggested in [31]. For these NLM-based filters, the decay parameter is determined using the rule-of-thumb, i.e., *h* = *β*·*σ*, where *β* and *σ* denote a predefined constant and the noise standard deviation [38], respectively. In these PCANet based NLM methods, the number of PCA filters in the two convolutional layers is fixed to be 12 and the patch size is 7×7, respectively.

### 3.1 The synthetic image

The experiment is conducted on the synthetic image corrupted by various levels of speckle noise with *σ* = 3, 4, 5, and 6, which is simulated based on Eq (1).

**Fig 4:**
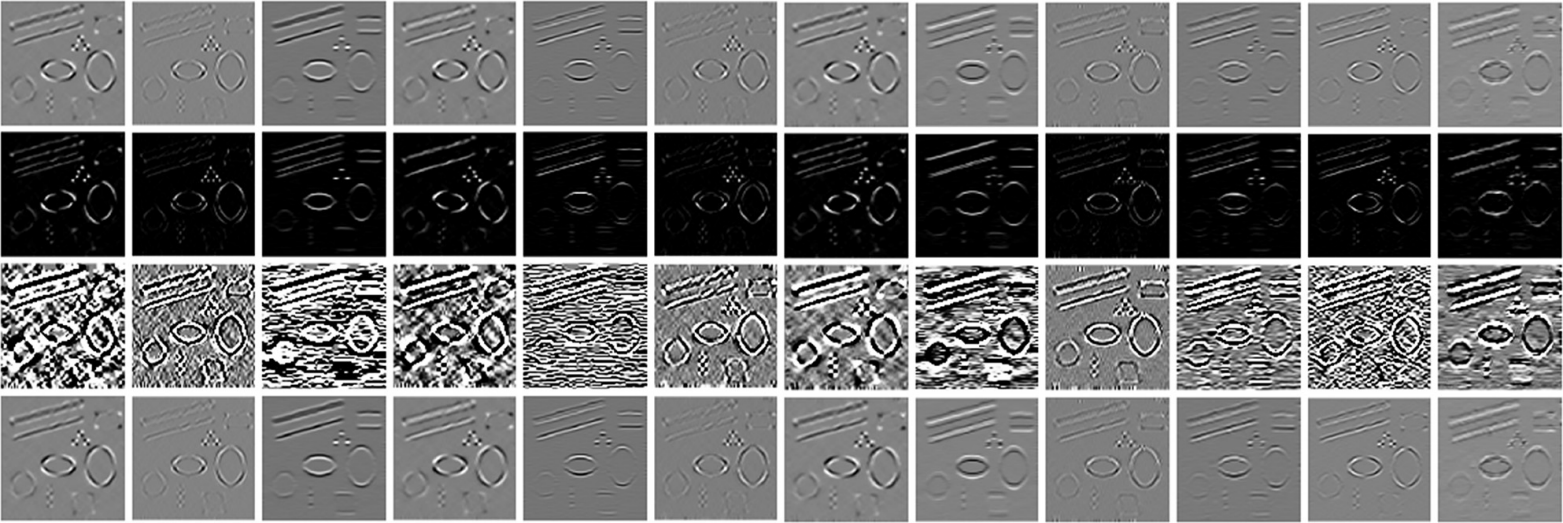
Comparisons of the learned feature images by various activation functions in the PCANet. The top row, the second row, the third row and the bottom row show the learned feature images by the original PCANet, ReLU-based PCANet, sigmoid-based PCANet and PReLU-based PCANet models, respectively.

Fig 4 shows the learned feature images by different activation functions based PCANet on the synthetic image with *σ* = 3. It can be seen from Fig 4 that the PReLU based PCANet model is more effective in learning the different features from the noisy image, which can facilitate the accurate computation of structural similarity between image patches.

**Fig 5:**
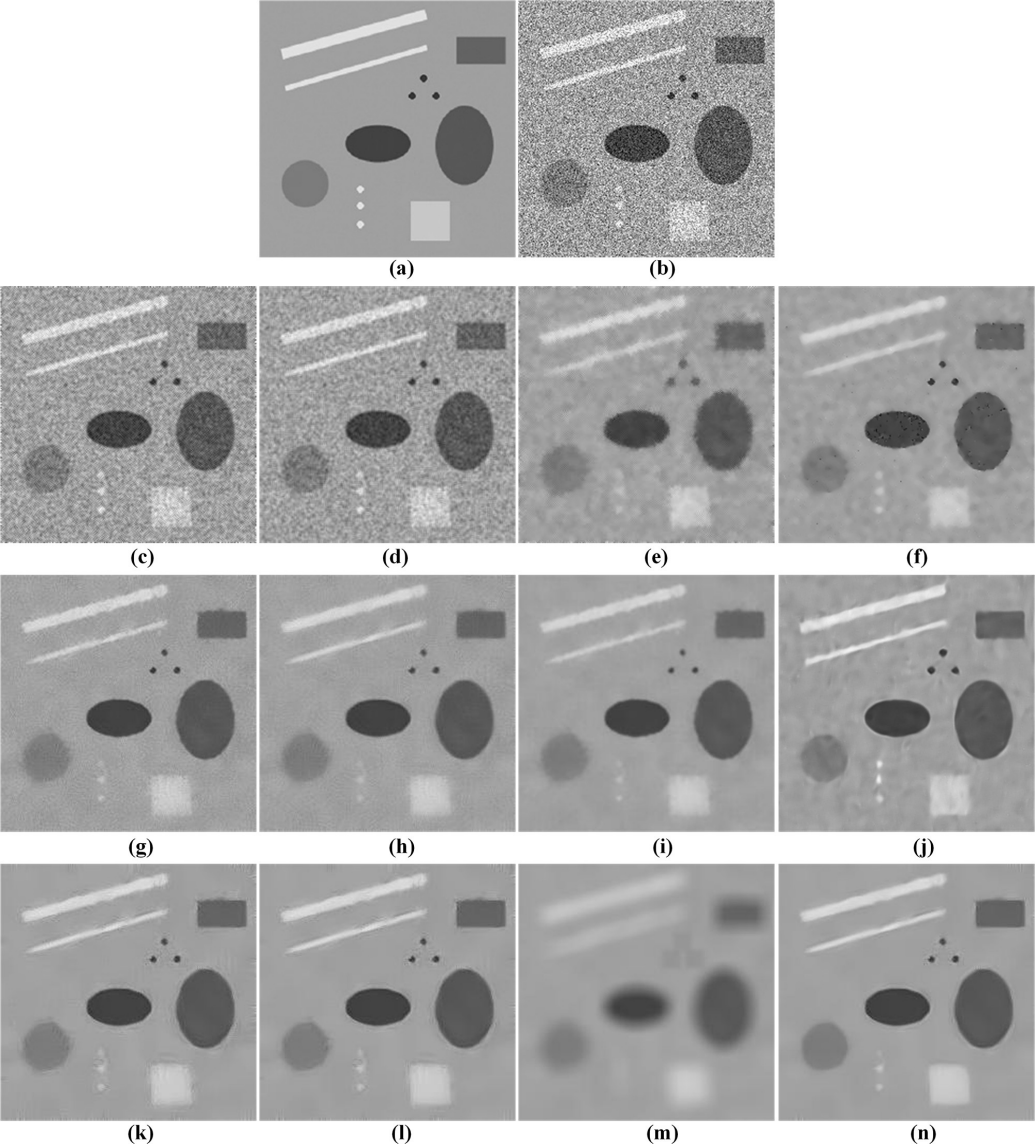
Visual comparison of restoration performance of the various despeckling methods on the synthetic image. (a) original image, (b) synthetic image with speckle noise (*σ* = 3), (c) Frost filter, (d) Kuan filter, (e) SBF filter, (f) SRAD filter, (g) TNLM filter, (h) OBNLM filter, (i) NLMLS filter, (j) DnCNN filter, (k) OPCA-NLM filter, (l) RPCA-NLM filter, (m) SPCA-NLM filter, and (n) PPCA-NLM filter.

Fig 5 presents a visual comparison of restored results for the different filters operating on the synthetic image. Obviously, speckle noise cannot be suppressed effectively by Frost and Kuan filter. In contrast, the SBF and SRAD filters perform better in noise removal, but they cause the blurring of image details. The evaluated NLM-based filters deliver sufficient speckle reduction. However, the TNLM and OBNLM filters produce the artifacts as shown in Fig 5(G) and 5(H), while the NLMLS filter damages some small image structure although it can reduce the artifacts to some extent. The DnCNN filter can facilitate enhancing the image contrast, but significant speckle noise and artifacts are observed in Fig 5(J). For the despeckling results based on PCANet methods, one can notice that there are obvious artifacts in Fig 5(K) and 5(L), whereas useful structure information cannot be preserved in Fig 5(M). By comparison, the PPCA-NLM method has better performance in removing speckle noise and avoiding artifacts.

**Fig 6:**
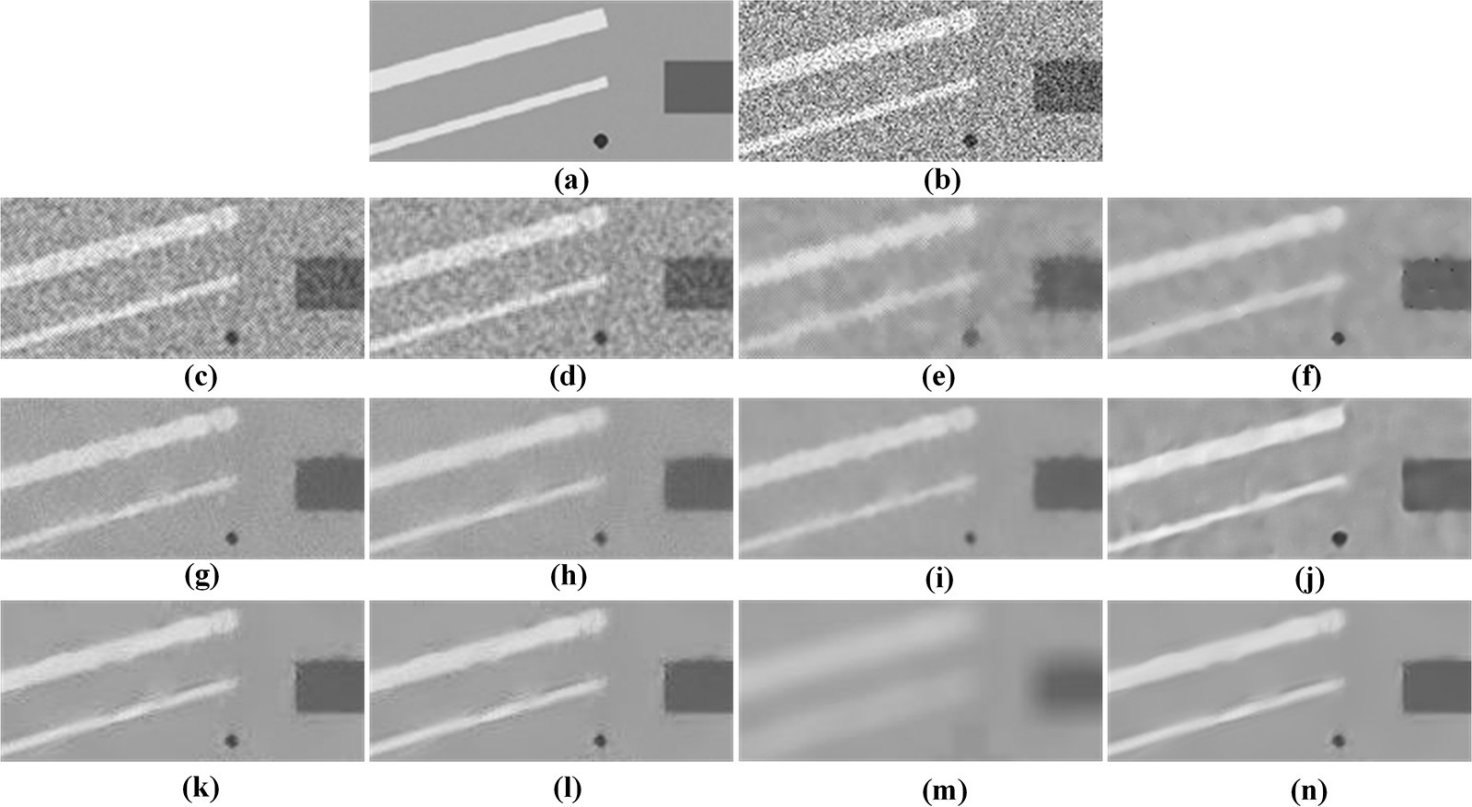
Comparison of zoomed details in Fig 5 for the twelve despeckling methods. (a) Original ROI, (b) Noisy ROI, (c) Frost filter, (d) Kuan filter, (e) SBF filter, (f) SRAD filter, (g) TNLM filter, (h) OBNLM filter, (i) NLMLS filter, (j) DnCNN filter, (k) OPCA-NLM filter, (l) RPCA-NLM filter, (m) SPCA-NLM filter, and (n) PPCA-NLM filter.

Furthermore, the zoomed views of a region of interest (ROI) from Fig 5 are shown in Fig 6 to demonstrate the advantage of the proposed method in preserving the small structures and edges. Clearly, Fig 6(C)–6(G) obtained with the Frost, Kuan, SBF, SRAD, and TNLM filters are unsatisfactory since these methods cannot provide good edge preservation. In Fig 6(H)–6(J), it can be seen that the OBNLM and NLMLS methods blur the edges and details of the rectangle, lines and circular point, while the DnCNN denoiser tends to distort the small structures and produce the obvious artifacts in the smooth region. Similarly, one can see from Fig 6(K)–6(M) that the OPCA-NLM and RPCA-NLM filters generate the blurred edges, while the SPCA-NLM filter causes serious damage to the image details. Compared with the other evaluated filters, the PPCA-NLM method is the most effective for preserving the fine structures and maintaining the edge sharpness in the image.

The quantitative evaluations are also made among all the tested methods. Two well-known evaluation indexes as peak signal-to-noise ratio (PSNR) and structural similarity index metrics (SSIM) [39] are used for performance appreciation, which are defined as:

$$\mathrm{PSNR}=10\;\log_{10}(\frac{255^{2}}{\frac{1}{W\times H}\sum_{i=1}^{W}\sum_{j=1}^{H}{\left(\hat{u}(i,j)-u(i,j)\right)}^{2}})$$ (Eq 8)

$$\mathrm{SSIM}=\frac{\left(2\mu_{\hat{u}}\mu_{u}+C_{1})(2\delta_{\hat{u}u}+C_{2}\right)}{\left(\mu_{\hat{u}}^{2}+\mu_{u}^{2}+C_{1})(\sigma_{\hat{u}}^{2}+\sigma_{u}^{2}+C_{2}\right)}$$ (Eq 9)

where *W* and *H* represent the width and height of the image, respectively. *u* is the noise-free image and $\hat{u}$ is the denoised image. $\mu_{\hat{u}}$ and *μ*_*u*_ are the mean intensity of images $\hat{u}$ and *u*, respectively. $\delta_{\hat{u}u}$ is the covariance between images $\hat{u}$ and *u*, $\sigma_{\hat{u}}$ and *σ*_*u*_ are the standard deviations of images $\hat{u}$ and *u*, respectively. *C*_1_ and *C*_2_ are the small constants to stabilize SSIM.

Tables 1 and 2 list the PSNR and SSIM values for all evaluated methods operating on the synthetic image corrupted with different levels of speckle noise, where the best values are marked in bold. It can be seen that the DnCNN can produce the maximum PSNR at all noise levels, and the PPCA-NLM method ranks the second. However, the proposed method provides significantly higher SSIM values than the DnCNN. The overall evaluation based on the above-mentioned visual inspection and the objective indexes demonstrates that the PPCA-NLM method exhibits the excellent despeckling performance for the synthetic image.

**Table 1:** The PSNR (dB) values of various despeckling methods on the synthetic image.

Methods	*σ* = 3	*σ* = 4	*σ* = 5	*σ* = 6
**Noisy image**	16.60	14.45	12.87	11.73
**Frost**	22.25	20.11	18.55	17.42
**Kuan**	26.03	23.79	22.11	20.99
**SBF**	27.23	25.88	24.72	23.95
**SRAD**	30.51	27.20	22.70	19.47
**TNLM**	29.96	27.70	26.28	25.12
**OBNLM**	30.50	28.44	27.26	26.24
**NLMLS**	30.96	28.91	27.70	26.50
**DnCNN**	**32.98**	**31.03**	**30.20**	**28.70**
**OPCA-NLM**	32.46	29.87	27.93	26.48
**RPCA-NLM**	32.59	30.30	28.54	27.10
**SPCA-NLM**	26.17	25.97	25.78	25.39
**PPCA-NLM**	**32.98**	30.79	29.30	27.71

**Table 2:** The SSIM values of various despeckling methods on the synthetic image.

Methods	*σ* = 3	*σ* = 4	*σ* = 5	*σ* = 6
**Noisy image**	0.105	0.071	0.054	0.042
**Frost**	0.254	0.185	0.146	0.119
**Kuan**	0.441	0.336	0.270	0.228
**SBF**	0.649	0.583	0.504	0.467
**SRAD**	0.863	0.698	0.415	0.219
**TNLM**	0.708	0.601	0.519	0.456
**OBNLM**	0.791	0.717	0.666	0.640
**NLMLS**	0.926	0.894	0.869	0.852
**DnCNN**	0.903	0.857	0.840	0.742
**OPCA-NLM**	0.927	0.851	0.751	0.703
**RPCA-NLM**	0.936	0.892	0.831	0.800
**SPCA-NLM**	0.830	0.829	0.828	0.818
**PPCA-NLM**	**0.950**	**0.928**	**0.904**	**0.879**

### 3.2 The simulated image

**Fig 7:**
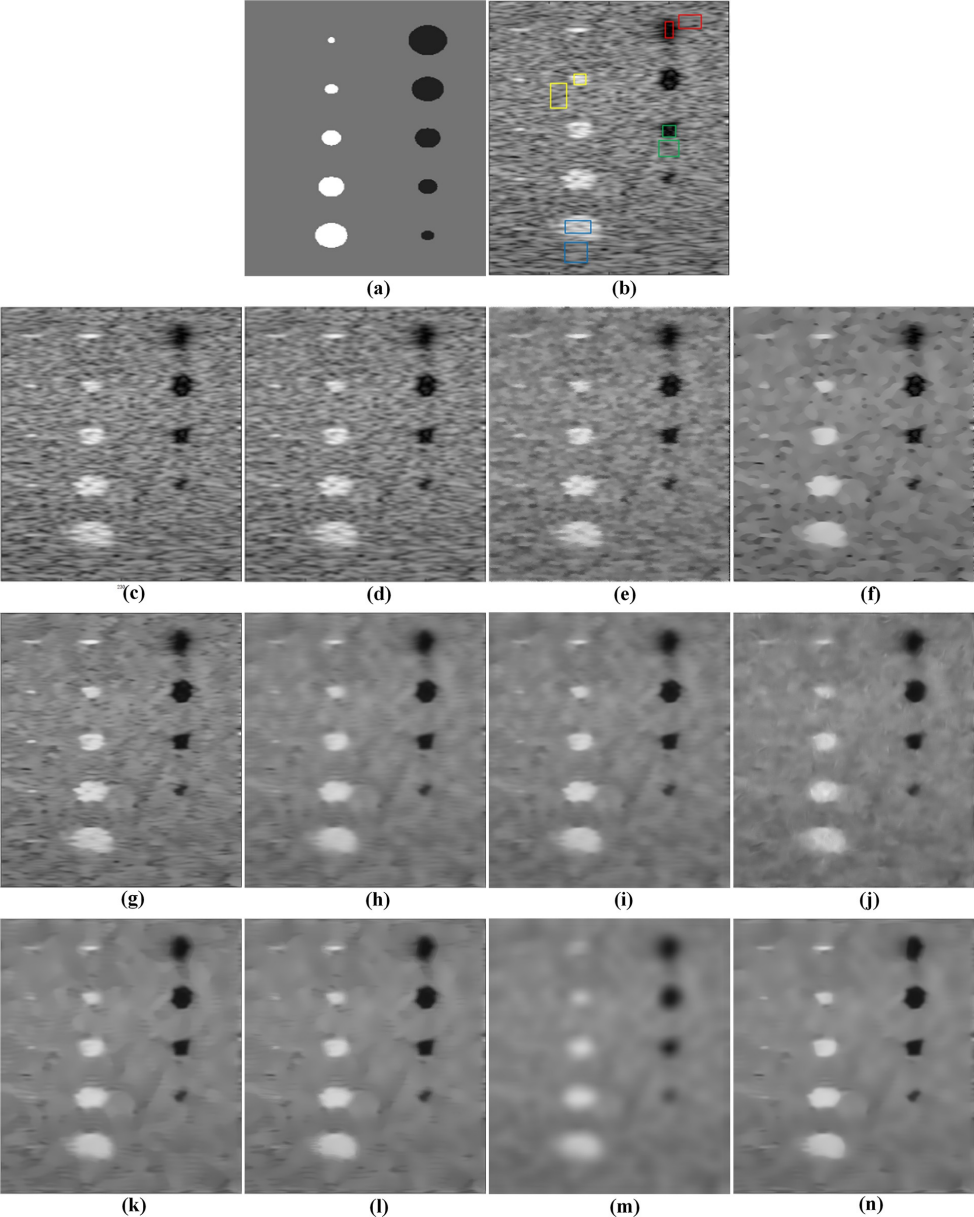
Visual comparison of restoration performance of various despeckling methods on the simulated image. (a) the “cyst” phantom, (b) simulated image generated by FieldⅡ and four ROIs marked with various colors, (c) Frost filter, (d) Kuan filter, (e) SBF filter, (f) SRAD filter, (g) TNLM filter, (h) OBNLM filter, (i) NLMLS filter, (j) DnCNN filter, (k) OPCA-NLM filter, (l) RPCA-NLM filter, (m) SPCA-NLM filter, and (n) PPCA-NLM filter.

A more challenging and relevant ultrasound image has been generated for the “cyst” phantom based on FieldⅡ simulation, which is a program to simulate ultrasound transducer fields and ultrasound imaging using the linear acoustics. The “cyst” phantom consists of a collection of point targets, five cyst regions, and five highly scattering regions [40]. Fig 7 presents the “cyst” phantom, the simulated image, and the despeckled results of all the tested methods. The Frost, Kuan and SBF filters keep much noise in the images as shown in Fig 7(C)–7(E). The SRAD filter performs well in noise removal, but it leads to the blurry edges and the staircase effect in the despeckled image. All NLM-based methods smooth out speckle noise more effectively than the above-mentioned local filters. However, the TNLM and NLMLS filters cannot maintain the sharpness of point targets and the OBNLM method produces the obvious artifacts. For the DnCNN method, many unwanted artifacts can be observed in the smooth region as shown in Fig 7(J). From Fig 7(K)–7(M), one can see that the OPCA-NLM and RPCA-NLM filters tend to retain speckle noise, while the SPCA-NLM filter produces an over-smoothed image. The comparison with the other eleven methods shows that the PPCA-NLM method can smooth out speckle noise well while providing the clearer boundaries of points as depicted in Fig 7(N).

Moreover, two other widely used evaluation indexes, i.e., equivalent number of looks (ENL) and contrast-to-noise ratio (CNR) [41] are utilized to quantitatively appreciate the despeckling performance, which are defined as:

$$\mathrm{ENL}=\frac{\mu_{b}^{2}}{\sigma_{b}^{2}}$$ (Eq 10)

$$\mathrm{CNR}=\frac{\left|\mu_{b}-\mu_{o}\right|}{\sqrt{\sigma_{b}^{2}+\sigma_{o}^{2}}}$$ (Eq 11)

where *μ*_*b*_ and *μ*_*o*_ are the mean intensity of background area and object area, *σ*_*b*_ and *σ*_*o*_ are the standard deviation of background area and object area, respectively.

Four pairs of ROIs are selected to evaluate the despeckling performance for these tested methods, and the ROIs are marked with different colors as shown in Fig 7(B). The ENL and CNR are listed in Tables 3 and 4, respectively. As regards ENL, the PPCA-NLM method provides the maximum values at all ROIs. Likewise, the proposed method performs the best for three ROIs except that its CNR is slightly less than that of the RPCA-NLM method for the blue ROI. The performance appreciation based on ENL and CNR confirms that the PPCA-NLM method is superior to other compared methods.

**Table 3:** The ENL values of four ROIs on the simulated ultrasound image.

Methods	ROI 1 (blue)	ROI 2 (green)	ROI 3 (yellow)	ROI 4 (red)
**Noisy image**	30.49	30.54	35.77	29.02
**Frost**	44.64	40.83	49.43	44.46
**Kuan**	49.10	43.82	54.18	50.10
**SBF**	88.05	63.55	120.95	170.36
**SRAD**	175.44	125.91	286.10	168.68
**TNLM**	148.97	162.10	186.82	185.09
**OBNLM**	282.94	439.41	417.05	483.60
**NLMLS**	279.34	510.83	430.73	529.97
**DnCNN**	160.93	550.15	449.86	800.90
**OPCA-NLM**	414.09	522.25	623.68	702.50
**RPCA-NLM**	520.27	595.68	935.03	850.07
**SPCA-NLM**	464.00	1110.27	1125.99	783.06
**PPCA-NLM**	**546.77**	**1336.78**	**1163.53**	**1145.27**

**Table 4:** The CNR values of four ROIs on the simulated ultrasound image.

Methods	ROI 1 (blue)	ROI 2 (green)	ROI 3 (yellow)	ROI 4 (red)
**Noisy image**	2.74	3.81	2.60	4.04
**Frost**	3.33	4.63	3.08	4.91
**Kuan**	3.51	4.82	3.23	5.18
**SBF**	4.67	6.06	3.97	7.38
**SRAD**	7.85	6.78	5.97	7.13
**TNLM**	6.81	9.99	5.12	9.66
**OBNLM**	9.34	14.29	5.58	13.20
**NLMLS**	9.40	14.34	5.83	13.18
**DnCNN**	6.75	14.72	4.40	14.13
**OPCA-NLM**	11.72	15.90	6.04	15.02
**RPCA-NLM**	**12.19**	16.39	6.52	15.69
**SPCA-NLM**	9.01	7.67	5.40	11.37
**PPCA-NLM**	11.45	**21.49**	**7.49**	**18.20**

### 3.3 The real ultrasound images

**Fig 8:**
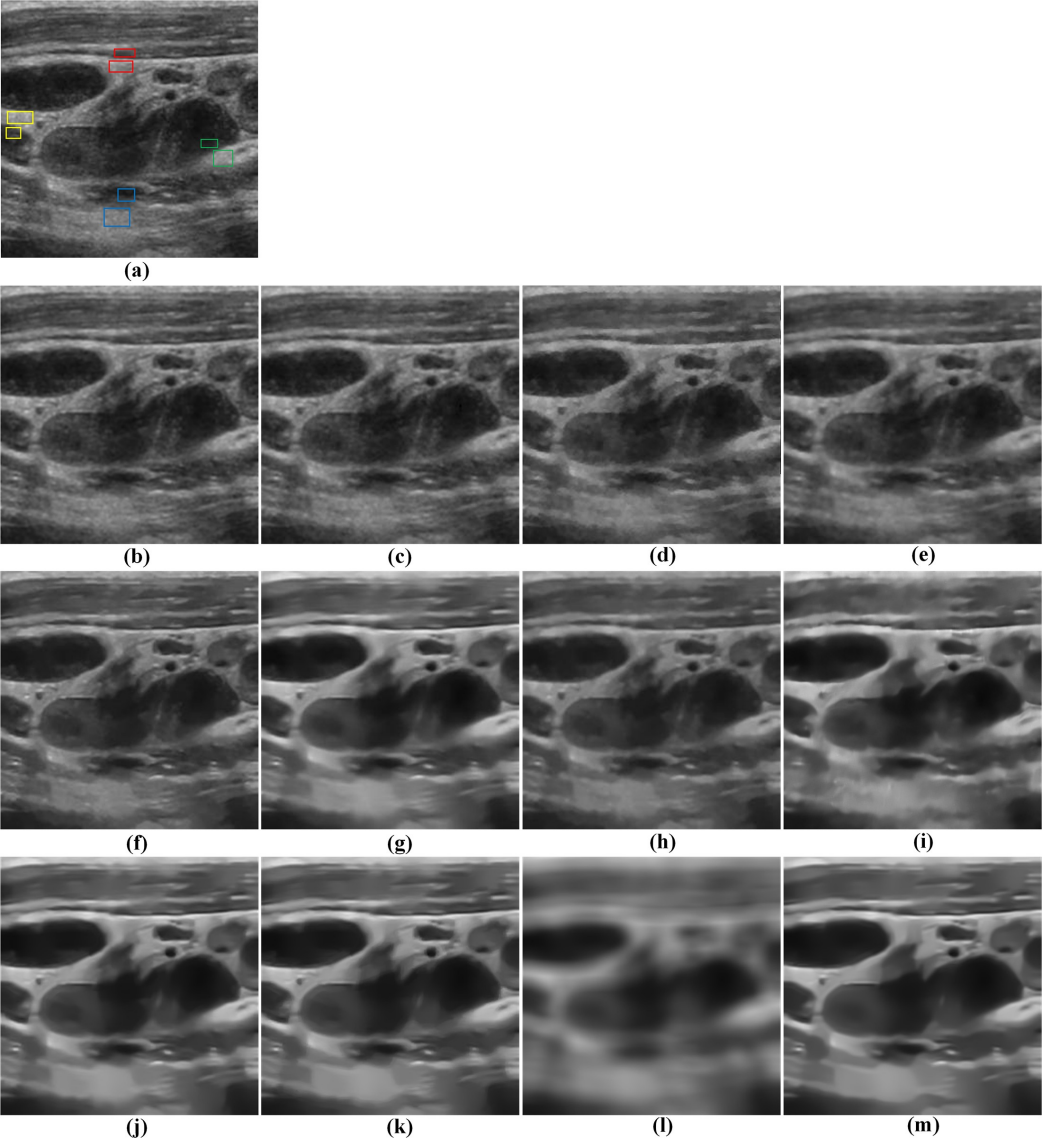
Comparison of restoration performance of various despeckling methods on a real ultrasound image of benign lymph nodes. (a) original noisy image and four ROIs marked with the different colors, (b) Frost filter, (c) Kuan filter, (d) SBF filter, (e) SRAD filter, (f) TNLM filter, (g) OBNLM filter, (h) NLMLS filter, (i) DnCNN filter, (j) OPCA-NLM filter, (k) RPCA-NLM filter, (l) SPCA-NLM filter, and (m) PPCA-NLM filter.

Three real ultrasound images are also used to further assess the effectiveness of the proposed method. Fig 8 shows the despeckled results for the compared methods on a real ultrasound image of benign lymph nodes. Visually, the Frost and Kuan filters maintain much speckle in the image as shown in Fig 8(B) and 8(C), while the SBF and SRAD filters result in the blurry object boundaries as shown in Fig 8(D) and 8(E). Compared with the TNLM and NLMLS filters, the PCANet based NLM filters are more effective in smoothing out speckle noise. However, the SPCA-NLM filter damages many image details. For the OBNLM and NLMLS filters, we can see from Fig 8(G) and 8(H) that they tend to generate the artifacts in some regions. By comparison, the PPCA-NLM method can preserve the sharpness of node boundaries better than the OBNLM and DnCNN methods. In contrast to the despeckled results of the OPCA-NLM and RPCA-NLM methods, the image details can be preserved with higher sharpness by the PPCA-NLM filter. The experiments on the real ultrasound image demonstrate the superiority of the proposed method to the other despeckling methods.

The quantitative results of four ROIs shown in Fig 8(A) are listed in Tables 5 and 6. With regard to ENL results, the PPCA-NLM method produces the maximum values at three ROIs among all the filters. In terms of CNR, the OPCA-NLM method provides the competitive results, followed by the PPCA-NLM and DnCNN methods. The above quantitative comparisons and visual inspection illustrate that the PPCA-NLM method is very suitable and practicable for despeckling the real ultrasound image.

**Table 5:** The ENL values of four ROIs on a real ultrasound image.

Methods	ROI 1 (blue)	ROI 2 (green)	ROI 3 (yellow)	ROI 4 (red)
**Noisy image**	219.45	338.21	112.34	102.96
**Frost**	333.15	569.40	172.76	136.67
**Kuan**	358.50	635.66	184.73	146.96
**SBF**	536.42	1155.25	245.41	153.03
**SRAD**	564.67	1145.40	292.45	193.45
**TNLM**	926.57	2204.59	243.73	191.82
**OBNLM**	1564.69	5393.52	591.38	396.95
**NLMLS**	1167.61	5212.99	391.28	290.55
**DnCNN**	1457.50	1419.98	534.73	538.04
**OPCA-NLM**	**2913.80**	6073.30	673.99	426.89
**RPCA-NLM**	2476.94	6232.09	687.21	487.45
**SPCA-NLM**	1857.40	413.23	666.11	282.31
**PPCA-NLM**	2054.56	**8175.66**	**845.83**	**935.00**

**Table 6:** The CNR values of four ROIs on a real ultrasound image.

Methods	ROI 1 (blue)	ROI 2 (green)	ROI 3 (yellow)	ROI 4 (red)
**Noisy image**	9.28	11.79	5.45	3.80
**Frost**	10.56	14.15	6.39	4.18
**Kuan**	10.71	15.04	6.51	4.27
**SBF**	12.63	17.52	7.27	4.38
**SRAD**	11.46	16.01	7.41	4.43
**TNLM**	15.83	19.90	7.36	4.79
**OBNLM**	18.29	21.53	9.78	6.21
**NLMLS**	14.65	20.97	8.32	5.44
**DnCNN**	16.88	18.01	**11.00**	5.12
**OPCA-NLM**	**19.86**	**21.88**	10.16	6.58
**RPCA-NLM**	19.45	21.22	10.08	6.67
**SPCA-NLM**	18.96	8.15	6.12	1.91
**PPCA-NLM**	19.42	21.80	10.33	**7.13**

**Fig 9:**
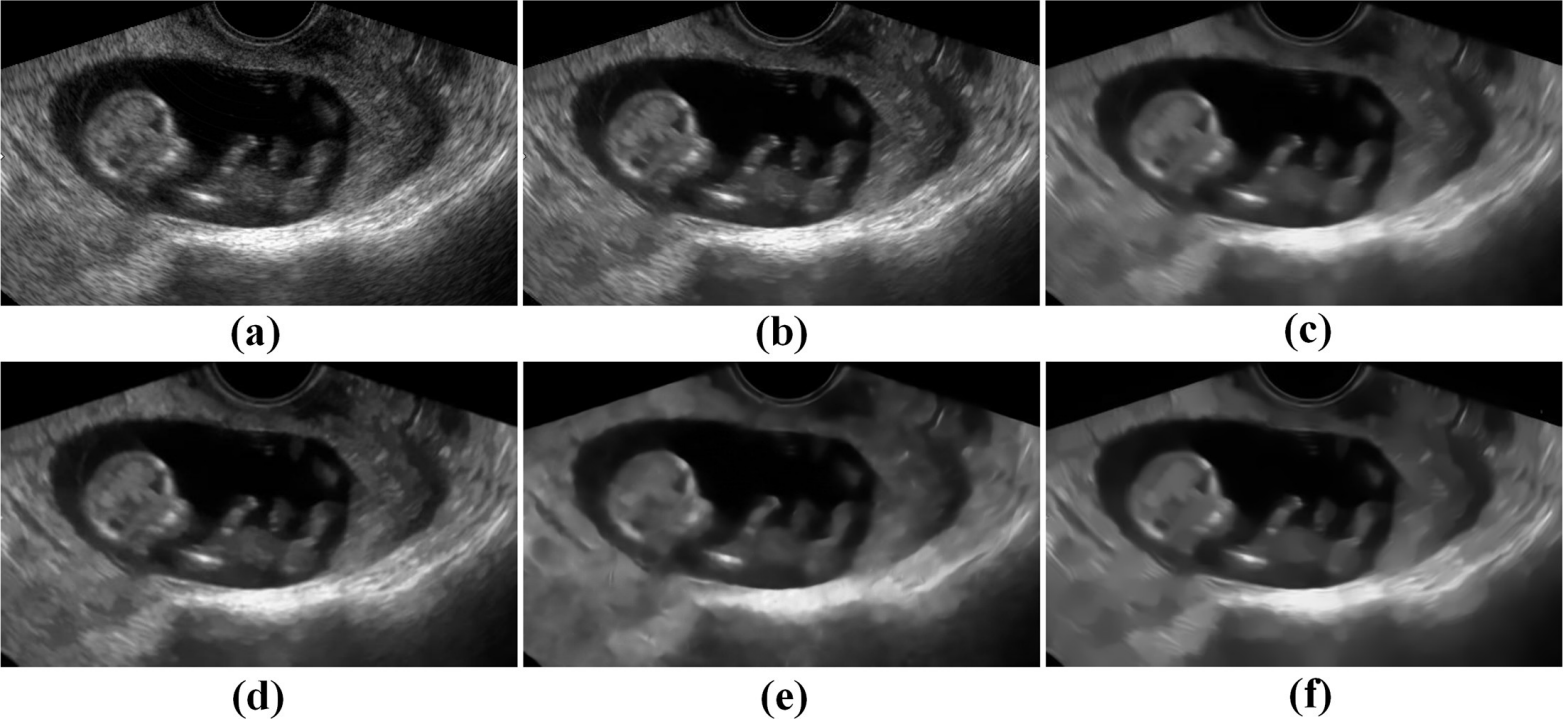
Comparison of restoration performance on a real fetal ultrasound image for the NLM-based and DnCNN methods. (a) original image, (b) TNLM filter, (c) OBNLM filter, (d) NLMLS filter, (e) DnCNN filter, and (f) PPCA-NLM filter.

**Fig 10:**
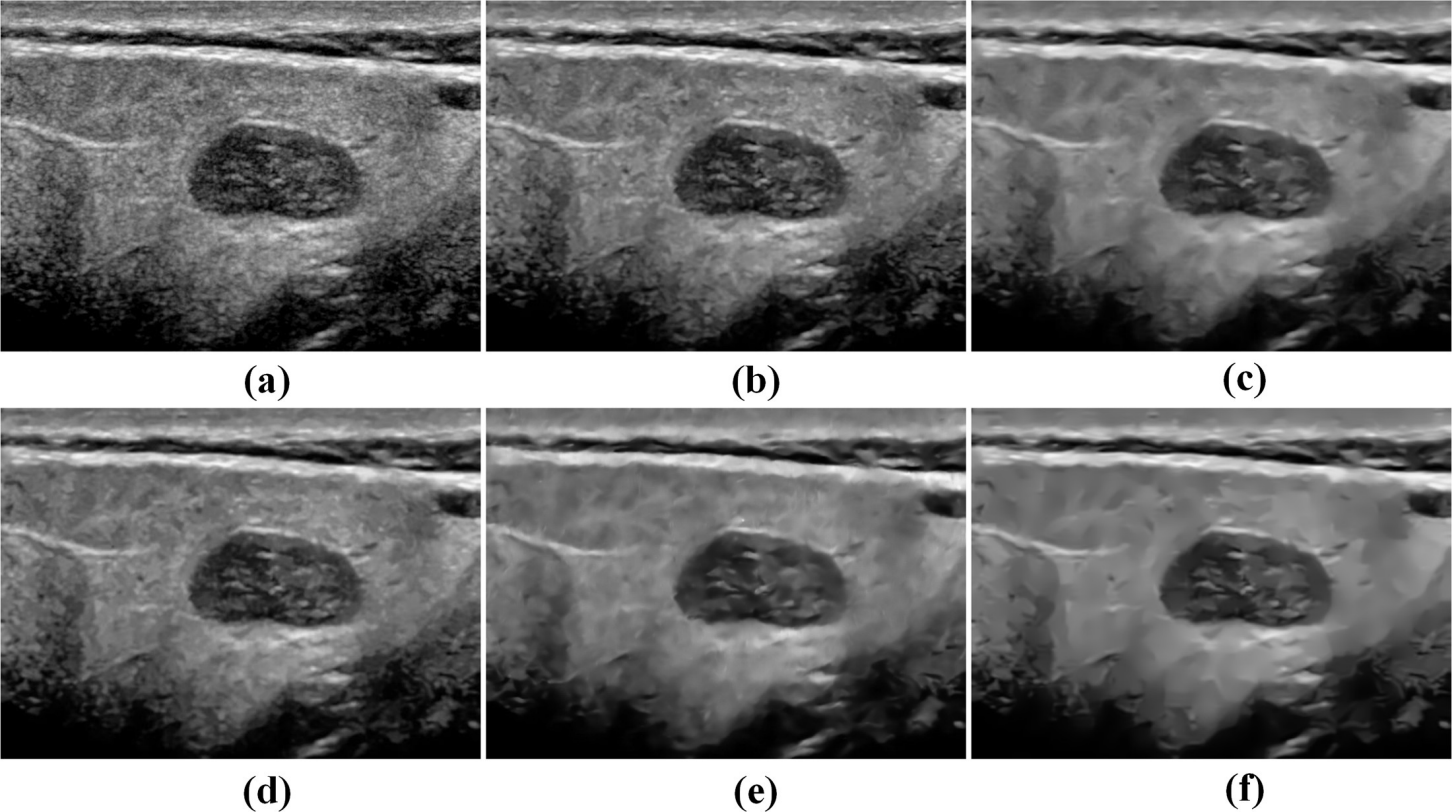
Comparison of restoration performance on a real parotid gland adenoma ultrasound image for the NLM-based and DnCNN methods. (a) original image, (b) TNLM filter, (c) OBNLM filter, (d) NLMLS filter, (e) DnCNN filter, and (f) PPCA-NLM filter.

Besides, two other clinical ultrasound images including the fetal image and the parotid gland image are used to further display the visual impression for the TNLM, OBNLM, NLMLS, DnCNN and PPCA-NLM filters. As indicated in Figs 9 and 10, the PPCA-NLM method outperforms the compared methods in that it not only effectively removes speckle noise but also enhances the sharpness of boundaries and retains image structures very well.

## 4 Conclusion

In this paper, the modified PCANet based deep learning baseline is introduced into NLM method for reducing speckle noise in the ultrasound images. This proposed method utilizes the intrinsic features extracted from an input noisy image by the PCANet to refine the similarity computation in the NLM method. The introduction of the intrinsic features instead of the gray-level information into the NLM method can facilitate the effective despeckling of ultrasound images due to its effectiveness in representing their structural information. The experimental results demonstrate that the proposed method outperforms the other compared methods in speckle reduction and image detail preservation. Therefore, the proposed approach has the great potential application to ultrasound-based clinical diagnosis. In future, the new deep learning models will be developed to denoise the images corrupted by Rician noise or Poisson noise.
